# Chronic Kidney Disease Itself Is a Causal Risk Factor for Stroke beyond Traditional Cardiovascular Risk Factors: A Nationwide Cohort Study in Taiwan

**DOI:** 10.1371/journal.pone.0036332

**Published:** 2012-04-30

**Authors:** Yi-Chun Chen, Yu-Chieh Su, Ching-Chih Lee, Yung-Sung Huang, Shang-Jyh Hwang

**Affiliations:** 1 Division of Nephrology, Department of Internal Medicine, Buddhist Dalin Tzu Chi General Hospital, Chiayi, Taiwan; 2 Division of Hematology-Oncology, Department of Internal Medicine, Buddhist Dalin Tzu Chi General Hospital, Chiayi, Taiwan; 3 Department of Otolaryngology, Buddhist Dalin Tzu Chi General Hospital, Chiayi, Taiwan; 4 Division of Neurology, Department of Internal Medicine, Buddhist Dalin Tzu Chi General Hospital, Chiayi, Taiwan; 5 School of Medicine, Tzu Chi University, Hualien, Taiwan; 6 Division of Nephrology, Department of Internal Medicine, Kaohsiung Medical University Hospital, Kaohsiung, Taiwan; FuWai Hospital, Chinese Academy of Medical Sciences, China

## Abstract

**Background:**

Cardiovascular disease (CVD) is a leading cause of mortality and morbidity in patients with chronic kidney disease (CKD). In Taiwan, CVD is dominated by strokes but there is no robust evidence for a causal relationship between CKD and stroke. This study aimed to explore such causal association.

**Methods:**

We conducted a nationwide retrospective cohort study based on the Taiwan National Health Insurance Research Database from 2004 to 2007. Each patient identified was individually tracked for a full three years from the index admission to identify those in whom any type of stroke developed. The study cohort consisted of patients hospitalized with a principal diagnosis of CKD and no traditional cardiovascular risk factors at baseline (*n* = 1393) and an age-matched control cohort of patients hospitalized for appendectomies (*n* = 1393, a surrogate for the general population). Cox proportional hazard regression and propensity score model were used to compare the three-year stroke-free survival rate of the two cohorts after adjustment for possible confounding factors.

**Results:**

There were 256 stroke patients, 156 (11.2%) in the study cohort and 100 (7.2%) in the control cohort. After adjusting for covariates, patients with primary CKD had a 1.94-fold greater risk for stroke (95% CI, 1.45–2.60; *p*<0.001) based on Cox regression and a 1.68-fold greater risk for stroke (95% CI, 1.25–2.25; *p* = 0.001) based on propensity score. This was still the case for two cohorts younger than 75 years old and without traditional cardiovascular risk factors.

**Conclusions:**

This study of Taiwanese patients indicates that CKD itself is a causal risk factor for stroke beyond the traditional cardiovascular risk factors. Primary CKD patients have higher risk for stroke than the general population and all CKD patients, irrespective of the presence or severity of traditional cardiovascular risk factors, should be made aware of the stroke risk and monitored for stroke prevention.

## Introduction

The prevalence of chronic kidney disease (CKD) is increasing rapidly throughout the world and cardiovascular disease (CVD) is a major cause of morbidity and mortality in patients with CKD [1]. However, the incidence of coronary heart disease and stroke is different for Western and Asian patients with CVD [2]. In Taiwan, stroke is more common than coronary heart disease in the context of CVD events [1].

There is conflicting evidence as to whether CKD is an independent risk factor for stroke. Several studies have indicated an elevated risk for stroke in CKD patients with pre-existing or co-existing traditional cardiovascular risk factors [3], [4], which suggests that these risk factors account for the additional stroke risk in CKD patients. However, large-scale population-based cohort studies have shown inconsistent results [5], [6], suggesting that CKD may simply be a marker for the duration or severity of traditional cardiovascular risk factors. CKD and stroke share traditional cardiovascular risk factors, such as advanced age, diabetes, hypertension, hyperlipidemia, and coronary heart disease [7]. Although most studies on the association between CKD and stroke risk have considered these vascular risk factors, none has adjusted for their duration and severity, limiting the extent to which these confounders can be fully adjusted [8]. This thus can result in residual confounding.

Moreover, meta-analysis based on observational studies cannot prove causality [8]. Although CKD is currently considered a non-modifiable entity [6], it is still unclear whether CKD per se, in the absence of traditional cardiovascular risk factors, independently increases the susceptibility to stroke. As stroke is a leading cause of mortality and morbidity worldwide, it is important to identify people at potential high risk so that stroke prevention therapy can be applied.

Taiwan National Health Insurance Research Database (NHIRD) is nationwide, population-based, reimbursement database that covers ∼99% of all Taiwan residents and provides comprehensive medical information. Therefore, these database have widely been used for epidemiological research [9]–[15], including studies on national CKD surveillance [1], [11]. Claims data can be used to accurately identify patients with CKD for study because of a high positive predictive value (97.5%) [16], [17]. Information on diagnoses and hospitalizations also has proven to be of high quality [9]–[15]. Most of the prior observational studies on the association between CKD and stroke risk have used Cox regression model or logistic regression, such that selection bias may exist in observational studies [18]. Propensity score analysis is now widely used in observational studies to reduce selection bias and identify true causal relationship [19]. The purpose of this study was to examine the causality between CKD itself and stroke based on the NHIRD from 2004 to 2007 by means of both Cox regression and propensity score methods.

## Materials and Methods

### Ethics Statement

This study was evaluated and approved by the NHIRD research committee and our institutional review board. Since all identifying personal information was stripped from the secondary files before analysis, the review board waived the requirement for written informed consent from the patients involved.

### Data Source

This study used data from the NHIRD released by the Taiwan National Health Research Institutes for the years 2004 to 2007. This dataset provided all original claims data from the Taiwan National Health Insurance program, a mandatory-enrollment, single-payment system that financed healthcare for all Taiwanese citizens (∼99% of the population) through a registry of board-certified specialists and contracted medical facilities [20]. According to a report of Taiwan's National Health Research Institutes, there were no significant differences in age, gender, or healthcare costs between this group and all enrollees [21].

### Study Population

This was a retrospective cohort study of patients aged at least 25 years old who were hospitalized with at least one principal diagnosis of CKD (ICD-9-CM codes 585) [11], [17] but without catastrophic illness registration cards for end-stage renal disease starting renal replacement therapy between January 1, 2004 and December 31, 2007. Only diagnoses given ahead of and in concurrence with the diagnosis of CKD were regarded as underlying comorbidities [11]. These comorbidities, namely traditional cardiovascular risk factors, include diabetes (ICD-9-CM codes 250), hypertension (ICD-9-CM codes 401–405), coronary heart disease (ICD-9-CM codes 410–414), and hyperlipidemia (ICD-9-CM codes 272–272.4), all of which can lead to CKD and stroke. Patients with preexisting or coexisting these risk factors and any type of stroke (ICD-9-CM codes 430–438) that were diagnosed before 2004 or during the index admission were excluded, thus creating a study cohort of primary CKD. A total of 1393 patients with primary CKD were identified. In Taiwan, a 5-stage CKD classification is defined according to estimated glomerular filtration rate sprouted in Taiwan since 2004, calculated by the simplified Modification of Diet in Renal Disease equation [22].

The control cohort included all patients aged at least 25 years old who were hospitalized for appendectomy as the primary procedure between January 1, 2004 and December 31, 2007 (ICD-OP code 47.0). Appendectomy patients were used as the control group because they had demographic and clinical characteristics similar to those of the general population [9], [10], [23], and because procedures related to appendectomy are not associated with increased risk of stroke and CKD. In the control cohort, patients with any type of stroke (ICD-9-CM codes 430–438), CKD (ICD-9-CM codes 585), catastrophic illness registration cards for end-stage renal disease starting renal replacement therapy before or during the index admission for appendectomy were excluded.

Advanced age was a major risk factor for CKD and stroke [7], so the study cohort was randomly age-matched with the control cohort (i.e., 25–44 yrs, 45–54 yrs, 55–64 yrs, 65–74 yrs, and older than 75 yrs) at a 1∶1 ratio to eliminate confounders. Each patient identified was individually tracked for full three years from the index admission to identify those in whom any type of stroke (ICD-9-CM codes 430–438) developed. Data on patient deaths were also examined for the period of 2004 to 2007 to calculate the stroke-free survival time, with case censored if the patient died from non-stroke causes.

The independent variables associated with stroke were gender, comorbidities (including diabetes, hypertension, coronary heart disease, and hyperlipidemia), geographic region of residence (divided as Northern, Central, Southern, and Eastern Taiwan) [24], urbanization level (urban, suburban, and rural) [25], and socioeconomic status [26]. Enrollee category [EC] was used as a proxy for socioeconomic status, and divided into four categories: EC 1 (highest socioeconomic status), EC 2, EC 3, and EC 4 (lowest socioeconomic status) [25].

### Statistical Analysis

All data were analyzed using the SAS statistical package (version 9.2; SAS Institute, Inc., Cary, N.C.) and SPSS (version 15; SPSS Inc., Chicago, IL, USA). A two-sided *p*-value<0.05 was considered statistically significant. A Pearson's *χ* ^2^ test was performed for categorical variables in the two cohorts, while the Kaplan-Meier method was used to estimate the three-year stroke-free survival rate. The cumulative risk of stroke was estimated as a function of time from initial treatment. The Cox proportional hazard regression model and the propensity score model were used to calculate the risk of the primary CKD group and the control group after adjustments for variables.

The propensity score model, as described by Rosenbaum and Rubin, was used to reduce the effect of selection bias in the two cohorts [27]. To obtain the propensity score in this study, patient characteristics were entered into a logistic regression model to predict selection for primary CKD. These characteristics were age, gender, comorbidities (i.e., diabetes, hypertension, coronary heart disease, and hyperlipidemia), geographic regions, urbanization level, and enrollee category (socioeconomic status). The study population was then stratified into five groups according to propensity score. The three-year cumulative risk of stroke was analyzed within each group and the Mantel-Haenszel odds ratio was estimated.

## Results

### Patient characteristics

The demographic characteristics and comorbidities of the primary CKD and control groups were shown in Table 1. Patients with primary CKD were more likely to reside in the suburban and rural areas and have lower socioeconomic status (EC 3 and 4) than patients in the control cohort. The control cohort had significantly more patients with diabetes, hypertension, and coronary heart disease, since the primary CKD group excluded patients with these comorbidities. Moreover, among the control cohort older than 75 years old (*n* = 507), 53 (10.5%) subjects had diabetes, 94 (18.5%) hypertension, 49 (9.7%) coronary heart disease, and 0 (0%) hyperlipidemia.

**Table 1:** Demographic characteristics and comorbidities of the primary chronic kidney disease (CKD) and control groups in Taiwan, 2004–2007 (n = 2786).

Variables	Primary CKD group (n = 1393) n (%)	Control group (n = 1393) n (%)	*p*
**Gender**			0.003
Male	657 (47.2)	735 (52.8)	
Female	736 (52.8)	658 (47.2)	
**Age, year**			NA
25–44	177 (12.7)	177 (12.7)	
45–54	196 (14.1)	196 (14.1)	
55–64	175 (12.6)	175 (12.6)	
65–74	338 (24.3)	338 (24.3)	
>75	507 (36.4)	507 (36.4)	
**Diabetes**			<0.001
Yes	0 (0.0)	118 (8.5)	
No	1393 (100)	1275 (91.5)	
**Hypertension**			<0.001
Yes	0 (0.0)	179 (12.8)	
No	1393 (100)	1214 (87.2)	
**Coronary heart disease**			<0.001
Yes	0 (0.0)	88 (6.3)	
No	1393 (100)	1305 (93.7)	
**Hyperlipidemia**			0.157
Yes	0 (0.0)	2 (0.1)	
No	1393 (100)	1391 (99.9)	
**Geographic region**			0.175
Northern	683 (49.0)	722 (51.8)	
Central	287 (20.6)	242 (17.4)	
Southern	386 (27.7)	391 (28.1)	
Eastern	37 (2.7)	38 (2.7)	
**Urbanization level**			0.024
Urban	322 (23.1)	384 (27.6)	
Suburban	587 (42.1)	544 (39.1)	
Rural	484 (34.7)	465 (33.4)	
**Enrollee category**			0.001
1–2	359 (25.8)	449 (32.2)	
3	678 (48.7)	609 (43.7)	
4	356 (25.6)	335 (24.0)	

Values are given as number (percentage).

### Analysis using Cox proportional regression model

**Figure 1:**
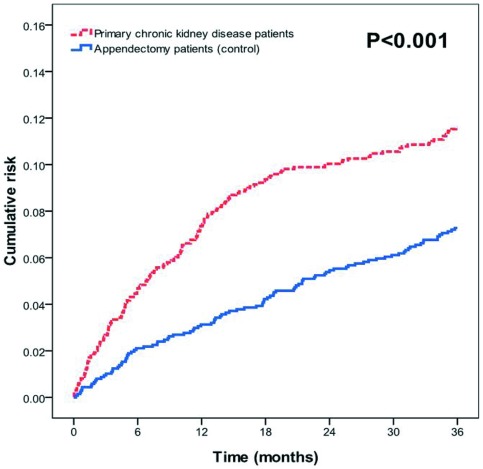
Cumulative risk of stroke for the primary chronic kidney disease and appendectomy (control) groups in the 3-year follow-up.

On the three-year follow-up, 156 patients (11.2%) in the primary CKD group and 100 patients (7.2%) in the control group had strokes (Table 2). Among patients older than 75 years old, 84 (16.6%) of the primary CKD group (*n* = 507) and 64 (12.6%) of the control group (*n* = 507) had strokes (*p* = 0.075, table not listed). Primary CKD patients had a higher cumulative risk of stroke than the control group (*p*<0.001, Fig. 1). The crude and adjusted hazard ratios for stroke in the primary CKD and control groups were shown in Table 2. After adjustments for age, gender, comorbidities, geographic region, urbanization level, and enrollee category, stroke hazard in the three-year follow-up period was 1.94-times higher (95% CI, 1.45–2.60; *p*<0.001) for the primary CKD group than the control group.

**Table 2:** Crude and adjusted hazard ratios for stroke among the primary chronic kidney disease (CKD) and control groups during the three-year follow-up period (n = 2786).

Development of Stroke	Control group (n = 1393) n (%)	Primary CKD group (n = 1393) n (%)	*p*
**3-Year follow-up period**			<0.001
Yes	100 (7.2)	156 (11.2)	
No	1293 (92.8)	1237 (88.8)	
**Age adjusted HR (95% CI)**	1	1.57 (1.22–2.02)	<0.001
**Adjusted HR (95% CI)** *	1	1.94 (1.45–2.60)	<0.001

Abbreviations: 95% CI = 95% confidence interval; HR = hazard ratio.

* Adjustments are made for patient's age, gender, diabetes, hypertension, coronary heart disease, hyperlipidemia, geographic region, urbanization level, and enrollee category.

### Analysis using propensity score

Stratification by propensity score and the three-year cumulative risk of stroke for the two cohorts were shown in Table 3. Based on the propensity model, the percentage of the control group decreased while the percentage of the primary CKD group increased from the first propensity to the fifth. When controlling for propensity in each of the five strata, the primary CKD group had a higher three-year cumulative risk of stroke (*p* = 0.001, Conchran-Mantel-Haenszel statistics) and 1.68-fold higher (95% CI, 1.25–2.25; *p* = 0.001) stroke odds than the control group. The three-year cumulative risk for stroke in the control group and the primary CKD group were 6.9% and 10.3% (*p*<0.001), respectively.

**Table 3:** Three-year cumulative risk of stroke among the control and primary chronic kidney disease (CKD) groups (n = 2786)^a^.

	Control group (n = 1393)			Primary CKD group (n = 1393)			
Propensity score stratum	No.	% of stratum	Cumulative risk (%)	No.	% of stratum	Cumulative risk (%)	*P*
1	458	82	10.5	99	18	4	0.048
2	276	50	1.8	281	50	7.2	0.002
3	242	43	7.6	316	57	10.5	0.215
4	221	40	6.4	336	60	12.5	0.019
5	196	35	8.2	361	65	17.3	0.003
Total	1393		6.9	1393		10.3	<0.001
							0.001^b^

a Stratum 1 had the strongest propensity for control; stratum 5, for primary chronic kidney disease.

b Conchran-Mantel-Haenszel statistics; adjusted odds ratio = 1.68, 95% confidence interval = 1.25–2.25.

### Analysis using Cox proportional regression model between two cohorts younger than 75 years old and without the above-mentioned comorbidities

We further examined the relationship of stroke risk between the study and control cohorts younger than 75 years old and without comorbidies (*n* = 1617) (Table 4). Among 1617 subjects, the control cohort had 731 subjects and the study cohort of primary CKD had 886 subjects. On the three-year follow-up, primary CKD patients still had a higher cumulative risk of stroke than the control cohort (*p*<0.001). Adjusted stroke hazard was 3.56-times higher (95% CI, 2.06–6.13; *p*<0.001) for the primary CKD group than the control cohort.

**Table 4:** Crude and adjusted hazard ratios for stroke among the primary chronic kidney disease (CKD) and control groups younger than 75 years old and without comorbidities during the three-year follow-up period (n = 1617).

Development of Stroke	Control group (n = 731) n (%)	Primary CKD group (n = 886) n (%)	*p*
**3-Year follow-up period**			<0.001
Yes	16 (2.2)	72 (8.1)	
No	715 (97.8)	814 (91.9)	
**Age adjusted HR (95% CI)**	1	3.54 (2.06–6.10)	<0.001
**Adjusted HR (95% CI)** *	1	3.56 (2.06–6.13)	<0.001

Abbreviations: 95% CI = 95% confidence interval; HR = hazard ratio.

* Adjustments are made for patient's age, gender, diabetes, hypertension, coronary heart disease, hyperlipidemia, geographic region, urbanization level, and enrollee category.

## Discussion

This study chose hospitalized patients with primary diagnoses of CKD and appendectomy as the study and control populations, respectively, from the NHIRD between 2004 and 2007. There are several reasons and strengths. First, the CKD Prevention Committee Taiwan Society of Nephrology was established in 2004 [28] for the high prevalence and low awareness of CKD and the low nephrology referral. Since 2004, nearly all hospitalized Taiwanese patients with a principal diagnosis of CKD received specialist consultation, thus reducing the probability of misdiagnosis. Second, appendectomy patients are suited as a surrogate of the general population because their difference is not discernible [9], [10], [23]. Third, the NHIRD has comprehensive health care information [29], including urbanization level and enrollee category that can be used as socioeconomic indicators for research to minimize the environmental effect [9], [10], [15], [30]. Furthermore, the NHIRD has highly acceptable validity for research in many diseases and stroke-risk associations [9], [10], [15], [20], [30] and provides sufficient statistical power to investigate the potential causal relationship of CKD itself on the risk of stroke.

To date, this is the first cohort study to demonstrate that CKD itself is a risk factor for stroke in the absence of traditional cardiovascular risk factors. The use of a population-based dataset has enabled the tracing of all stroke events within a three-year follow-up and allowed the exclusion of patients with preceding or concurrent traditional risk factors for stroke. After adjustment for covariates, the results indicate that, relative to the control cohort, hospitalized primary CKD patients have a stroke risk that is 1.94-fold higher by Cox proportional regression analysis and 1.68-fold higher by propensity analysis during a three-year follow-up. This was still the case for two cohorts younger than 75 years old and without traditional cardiovascular risk factors. This suggests that all patients with CKD, irrespective of the presence or extent of traditional cardiovascular risk factors, should be monitored to prevent stroke. These findings have important implications in future CKD prevention care programs in Taiwan, which has an increasing number of CKD patients, a current CKD prevalence of 11.9% [31], and a population in which 30% of CVD events are accompanied by stroke [1], as well as for Asians because Asian CKD patients are at higher risk of stroke than non-Asians [8].

Two methods have been used to validate the association of primary CKD and stroke events: the Cox proportional regression hazards model and the propensity score model. Selection bias can occur in Cox regression analysis [18], so the propensity score method developed by Rosenbaum and Rubin [27] was also used to minimize selection bias [19]. The propensity score strata provides similar distributions of possible confounders in the study and control groups, and removes more than 90% of overt bias from confounders [32]. It has also been reported that propensity score analysis may attenuate the bias of odds ratios toward the null relative to conventional regression adjustment [32]. In the current study, the propensity score results indicate a 1.68-fold higher risk for stroke in CKD patients, very close to the Cox regression results.

All previous cohort [3]–[6], [33]–[38] and cross-sectional [39], [40] studies of stroke risk and CKD have targeted selected populations with co-existing traditional cardiovascular risk factors at baseline, and used the Cox proportional regression hazards model to evaluate the association. However, these studies have had discordant results. Results of the current study differ from those of previous population-based studies [5], [6] wherein mild and moderate CKD is not an independent risk factor for CVD in the generation population. Although the cohorts of these two previous studies have been followed-up for more than 10 years, traditional cardiovascular risk factors are not uniformly distributed in the CKD and control groups, so the reported relationship of CKD and CVD risk may have been biased by the co-existence of CKD with traditional cardiovascular risk factors. The presence of CKD may simply indicate the duration or severity of these factors.

On the other hand, results of the current study are consistent with those of several other previous studies [3], [4], [33]–[36] which reported CKD as an independent risk factor for stroke following adjustments for traditional cardiovascular risk factors. Although such adjustments do not remarkably change this association in these previous studies, the results may imply that CKD itself is a causal factor in the pathogenesis of stroke, or that CKD is simply a marker of cerebral small vessel pathology. The kidney and brain share unique susceptibilities to vascular injury, so some vascular risk factors like hypertension, diabetes, and heart disease may cause similar vascular injuries in both organs [39]–[41].

Furthermore, the adjusted estimates of stroke risk in CKD may reflect residual confounding from traditional cardiovascular risk factors if CKD is simply a marker for the duration and severity of these factors. For instance, hypertension is a powerful risk factor for stroke in CKD [8]. The current study makes up for these deficiencies and clearly indicates that CKD itself independently contributes to the development of stroke.

The pathogenic mechanisms involved in this association may be related to non-traditional risk factors, such as endothelial dysfunction, oxidative stress, inflammation, hyperhomocysteinemia, and thrombogenic factors [4], [38], [42]. All of these factors may play a role in accelerated atherosclerosis in the arteries of both the kidney and brain, making CKD a non-modifiable entity [6]. This indicates the importance of active and early detection of undiagnosed CKD, irrespective of the levels of traditional cardiovascular risk factors, and the need for future interventional studies to identify non-traditional risk factors so that more preventive measures can be implemented. In Taiwan, the CKD Preventive Project had extended to care patients of CKD stage 1–3 since 2011, which may be helpful in identifying potential subjects at high risk for future stroke.

This study has some limitations. First, the diagnoses of CKD, stroke, appendectomy, and other comorbidities are completely based on ICD codes, and misclassification is possible. However, such misclassification was likely to be random and thus would tend to underestimate rather than overestimate the association [11], [13]. Furthermore, the National Health Insurance Bureau of Taiwan has randomly reviewed the charts and audited all medical charges, and given heavy penalties for outlier charges or malpractice, thus data quality is worthwhile trusting. Second, individual information such as blood pressure level, laboratory data, as well as stage, duration, and etiology of CKD was not available through administrative data of the NHIRD. Previous studies have documented the similarity on the diagnosis of CKD between by claims data in large administrative data sets and by estimated glomerular filtration rate in the general population [11], [43]. Furthermore, the concept that CKD is divided into 5 stages sprouted since 2004 in Taiwan, thus the primary CKD group in the present study should comprise all stages. Third, the type and severity of strokes cannot be extracted from ICD codes. Nonetheless, hospitalized Taiwanese patients with principal diagnoses of stroke are routinely given brain CT imaging, thus reducing the probability of misdiagnosis. Fourth, individual information such as smoking, alcohol consumption, family history of stroke, body mass index, dietary habits, and physical activity, all of which may contribute to stroke [7], was not available in the NHIRD. Therefore, the association between CKD itself and stroke may be partially explained by residual confounding by these factors. However, these factors contribute far less to stroke than traditional cardiovascular risk factors, and that their inclusion will unlikely influence the results [14]. Moreover, the increased risk of stroke among subjects with a positive family history of stroke is related to the expression of genetic heritability of stroke risk factors, a shared environment such as lower physical activity and socioeconomic status, or both [15], [44]. The urbanization standards of Taiwan's National Health Research Institute include education level. Therefore, we simultaneously took urbanization level and enrollee category as socioeconomic indicators in the model to minimize the environmental effect and confounding effect of family history of stroke [15]. Further study linking administrative data and primary hospitalization information is worthy of future investigation. Nonetheless, given the magnitude and statistical significance of the observed effects in this study, these limitations are unlikely to comprise the results. Finally, as with any observational study, potential unmeasured confounders may exist in the cases and controls [13].

In conclusion, this cohort study is the first to validate a clear relationship between CKD per se and stroke in the absence of traditional cardiovascular risk factors. Using two forms of statistical analysis, Cox regression and propensity score models, the results consistently indicate that patients with primary CKD have increased risk for stroke within three years independent of traditional cardiovascular risk factors. Therefore, all CKD patients, even in the absence of traditional cardiovascular risk factors, should be considered at risk for stroke and be aggressively managed for stroke prevention. Moreover, public awareness about the increased risk of stroke in CKD should be actively promoted.
